# Risk of detrimental recommendations for cancer pain management

**DOI:** 10.1186/s12967-021-02831-4

**Published:** 2021-04-20

**Authors:** Marco Maltoni, Romina Rossi

**Affiliations:** Palliative Care Unit, Istituto Scientifico Romagnolo per lo Studio e la Cura Dei Tumori (IRST) IRCCS, via P. Maroncelli 40, Meldola, FC Italy

Dear Editor,

We read with interest the article "The Role of Opioids in Cancer Response to Immunotherapy" by Botticelli et al. [1], a retrospective study that analyzes the clinical outcomes of cancer patients treated with immunotherapy, some of them also treated with different supportive/palliative drugs, including opioids.

Some of the authors' comments on their results have raised some questions in us, which we express in this letter.

The major findings of the study were the following.

Multivariate Analysis (MA) showed that Performance Status negatively affected Overall Survival (OS), while no drug did not affect that. However, opioids had a negative correlation with Progression Free Survival (PFS) at the MA, and all other supportive/palliative drugs had a negative impact on PFS and OS in the Univariate Analysis (UA) in those patients with an ECOG PS ≥ 1 and in patients with a high tumor burden. No data on Health Related Quality of Life are reported.

Based on these data, the Authors' comments were, in our opinion, very “strong”. In particular, at the end of "Introduction", the Authors state: *"The removal of concomitant drugs with immunoinhibitory action could play a decisive role in restoring the response to immunotherapy treatment, thus improving patient outcomes".*

From this and other phrases in the “discussion” session, we fear that some patients will no more be treated with drugs useful for their needs.

The statement seems imprudent to us for at least three reasons.

The first. Since this was not a randomized clinical trial, it was not possible, nor was it carried out by the authors, any outcome comparison between patients treated versus not treated with a certain drug.

The second. Although the study is very interesting, it has a number of limitations, correctly reported by the Authors: retrospective nature, heterogeneous population in terms of primary site, immuno- and supportive therapies.

But an even more fundamental reason (the third) could make their comment too firm. The supportive and palliative drugs were not subministered randomly, but to patients in clinical situations that required supportive/palliative treatments.

However, and this is our main point, those clinical settings are "per se" detrimental in terms of quality and/or quantity of life.

Fungal or bacterial infections have a negative impact on quantity of survival [2, 3]. Pain is harmful on the quality of survival, but also on the quantity, due to the immunosuppressive effect of severe pain itself [4–6]. Dyspnea or cachexia-anorexia cancer syndrome (CACS), two possible reasons of glucocorticoids administration, negatively impact quality and quantity of survival [7, 8].

So, how could the quantity and quality of survival of patients taking those drugs be compared with those items of the same patients (with the problems that had the indication for taking those drugs) without taking the drugs? In other words, how would it be possible to measure the “net” result of the administration of palliative drugs versus the natural course of certain symptoms or syndromes avoiding due treatments? That is, what about quality and quantity of life of the patients with bacterial or fungine infections or severe pain or dyspnea or CACS, with their “natural history”, without the drugs to keep the clinical situations controlled?

No one will ever know whether the removal of certain drugs (whose alternatives, if any, are less effective) administered for specific clinical reasons would lead to an improvement (as the Authors claim) or a worsening of patients’ outcomes.

We assume it will never be possible to make this theoretical comparison.

Therefore, we consider appropriate a more cautious conclusion, such as that of Boland et al., at the end of their systematic review concerning the effect of opioids on immunomodulation: *"Judicious doses of opioids should continue to be used as part of a multimodal approach for the management of patients with cancer pain"* [9].

## Data Availability

Not applicable.
